# Space Rather than Seasonal Changes Explained More of the Spatiotemporal Variation of Tropical Soil Microbial Communities

**DOI:** 10.1128/spectrum.01846-22

**Published:** 2022-11-23

**Authors:** Yaqing Wei, Fei Quan, Guoyu Lan, Zhixiang Wu, Chuan Yang

**Affiliations:** a Rubber Research Institute, Chinese Academy of Tropical Agricultural Sciences, Haikou City, Hainan Province, People’s Republic of China; b School of Life Sciences, Fudan University, Shanghai, People’s Republic of China; c Hainan Danzhou Tropical Agro-ecosystem National Observation and Research Station, Danzhou City, Hainan Province, People’s Republic of China; d College of Ecology and Environment, Hainan University, Haikou, China

**Keywords:** rubber plantation, rainforest, soil microbiome, community structure, diversity, drivers

## Abstract

Soil microbiomes play an essential role in maintaining soil geochemical cycle and function. Although there have been some reports on the diversity patterns and drivers of the tropical forest soil microbial community, how space and seasonal changes affect spatiotemporal distribution at the regional scales are poorly understood. Based on 260 soil samples, we investigated the spatiotemporal patterns of rubber plantations and rainforest soil microbial communities across the whole of Hainan Island, China during the dry and rainy seasons. We examined soil bacterial and fungal composition and diversity and the main drivers of these microbes using Illumina sequencing and assembly. Our results revealed that the diversity (both alpha and beta) spatiotemporal variation in microbial communities is highly dependent on regional location rather than seasonal changes. For example, the site explained 28.5% and 37.2% of the variation in alpha diversity for soil bacteria and fungi, respectively, and explained 34.6% of the bacterial variance and 14.3% of the fungal variance in beta diversity. Soil pH, mean annual temperature, and mean annual precipitation were the most important factors associated with the distribution of soil microbial communities. Furthermore, we identified that variations in edaphic (e.g., soil pH) and climatic factors (e.g., mean annual temperature [MAT] and mean annual precipitation [MAP]) were mainly caused by regional sites (*P* < 0.001). Collectively, our work provides empirical evidence that space, rather than seasonal changes, explained more of the spatiotemporal variation of soil microbial communities in tropical forests, mediated by regional location-induced changes in climatic factors and edaphic properties.

**IMPORTANCE** The soil microbiomes communities of the two forests were not only affected by environmental factors (e.g., edaphic and climatic factors), but also by different dominant geographic factors. In particular, our work showed that spatial variation in bacterial and fungal community composition was mainly dominated by edaphic properties (e.g., pH) and climatic factors (e.g., MAT and MAP). Moreover, the environmental factors were mainly explained by geographic location effect rather than by seasonal effect, and environmental dissimilarity significantly increased with geographic distance. In conclusion, our study provides solid empirical evidence that space rather than season explained more of the spatiotemporal variation of soil microbial communities in the tropical forest.

## INTRODUCTION

Soil microorganisms play a critical role in the functioning of ecosystems around the world. They are known to maintain the rates and stability of multiple ecosystem processes (1, 2). Therefore, we must develop a better understanding of the distribution and ecological drivers of belowground communities. There have been reports in the literature on the drivers of tropical forest soil microbial communities (2–7). A study by Lan et al. (4) showed that variation of soil microbial communities of rubber plantations was mainly caused by historical contingencies, whereas seasonal changes explained variation at the local scale. However, the relative importance of spatial heterogeneity versus time for driving soil microbial community composition at the regional scale is poorly understood.

Previous studies have investigated the soil microbial communities associated with diverse forests. Many published reports have examined the response of microbial composition to spatial or temporal change (3, 8–14). Biogeography, geographic location, and seasonal change have been demonstrated to influence community composition. However, the extent to which each variable plays a role has differed between studies (2–4, 15, 16). Most studies on soil microbial ecology have focused on temperate trees, crops, or other plant species (17–19), providing limited insight into the tropical forest, specifically rubber plantations and tropical rainforests. Moreover, these examples have shown that soil microbial communities are influenced by spatial or temporal change; however, understanding of how seasonal changes (dry and rainy seasons) affect the composition and diversity of soil microbial communities at the regional scale is still limited.

Hainan Island is a part of the Indo-Burma, a global biodiversity hot spot (20, 21), which is home to a large area of tropical rainforest, accounting for 17.3% of the island’s area (5). During the last two decades, rubber tree plantations seem to have multiplied quickly throughout Southeast Asia (22). Tropical rubber tree plantations have become the most economically important vegetation ecosystem in tropical China, particularly on Hainan Island (23). Now, rubber forests account for almost 25% of the total vegetation area on Hainan Island (24). Soil microbes play important functional roles in tropical forest ecosystems. Tropical rainforest and rubber plantations are the two most important terrestrial ecosystems in Hainan. Therefore, it is necessary to study the distribution patterns and ecological drivers of the soil microbial communities of these two forests.

Hainan Island is the largest tropical area with the lowest latitude in China (25), with an evident dry and rainy season within a year. Previous work on the island has shown that seasonal change and site location were the dominant factors resulting in shifts in bacterial composition at the local and geographic scales, respectively (3, 4). However, previous studies of soil bacterial diversity have been limited, particularly in the sample sizes and sampling scales they used (3–5). Moreover, little attention has been paid to the relative contributions of seasonal change and spatial heterogeneity in shaping the distribution of soil microbial communities. Other research has shown that the dominant phylum of a soil fungal community was mainly correlated with slow-changing environmental factors (e.g., pH and temperature) (5), which were influenced by the historical contingency of the geographic location (26, 27). Therefore, we hypothesize that space or site location would explain more of the spatiotemporal variation in tropical soil microbial communities than seasonal change at the regional scale.

To test this hypothesis, we evaluated soil microbial composition and diversity patterns based on field samples of rubber and rainforest plantations during both the dry and rainy season across the whole of Hainan Island (Fig. S1). The primary goals of this study were to (i) identify the taxa composition and spatial patterns (including alpha and beta diversity) of bacterial and fungal communities in the soil across different seasons; (ii) quantify the drivers of spatiotemporal variation in the soil microbiome, including the relative influence of seasonal changes, edaphic characteristics, climatic variables, and geographic distance; and (iii) quantify the main predictors of the soil microbial communities of tropical forests.

## RESULTS

### Environmental variables and community composition.

For the 12 measured environmental variables, respective variation was mainly explained by geographic sites: water content (WC), soil organic matter (SOM), soil pH, nitrate nitrogen (NN), available potassium (AK), total potassium (TK), mean annual precipitation (MAP) and mean annual temperature (MAT) were highly localized; site location had a stronger effect than season on environmental variables (Fig. S2 and S3). Furthermore, environmental dissimilarity (environmental distance between sites based on measured environmental variables) significantly increased with geographic distance during both the dry and rainy seasons (Fig. S4).

At a 97% taxonomy identity threshold, a total of 14,834 operational taxonomic units (OTUs) were detected for the fungal communities and 13,294 for the bacterial communities. In the rubber plantation, most bacterial OTUs were assigned to Proteobacteria (33.28%), Acidobacteria (19.27%), Chloroflexi (13.63%), and Actinobacteria (12.41%) at the phylum level (Fig. S5a); and the fungal OTUs were assigned to Sordariomycetes (13.63%), Agaricomycetes (12.20%), and Eurotiomycetes (4.42%) at the class level (Fig. S5c). In the rainforest, most bacterial OTUs were assigned to Proteobacteria (43.13%), Acidobacteria (20.30%), and Actinobacteria (10.24%) at the phylum level (see Fig. S5b); and fungal OTUs were assigned to Agaricomycetes (30.13%), Tremellomycetes (18.69%) and Sordariomycetes (12.14%) at the class level (Fig. S5d).

### Spatiotemporal patterns of the soil microbiome community.

**FIG 1:**
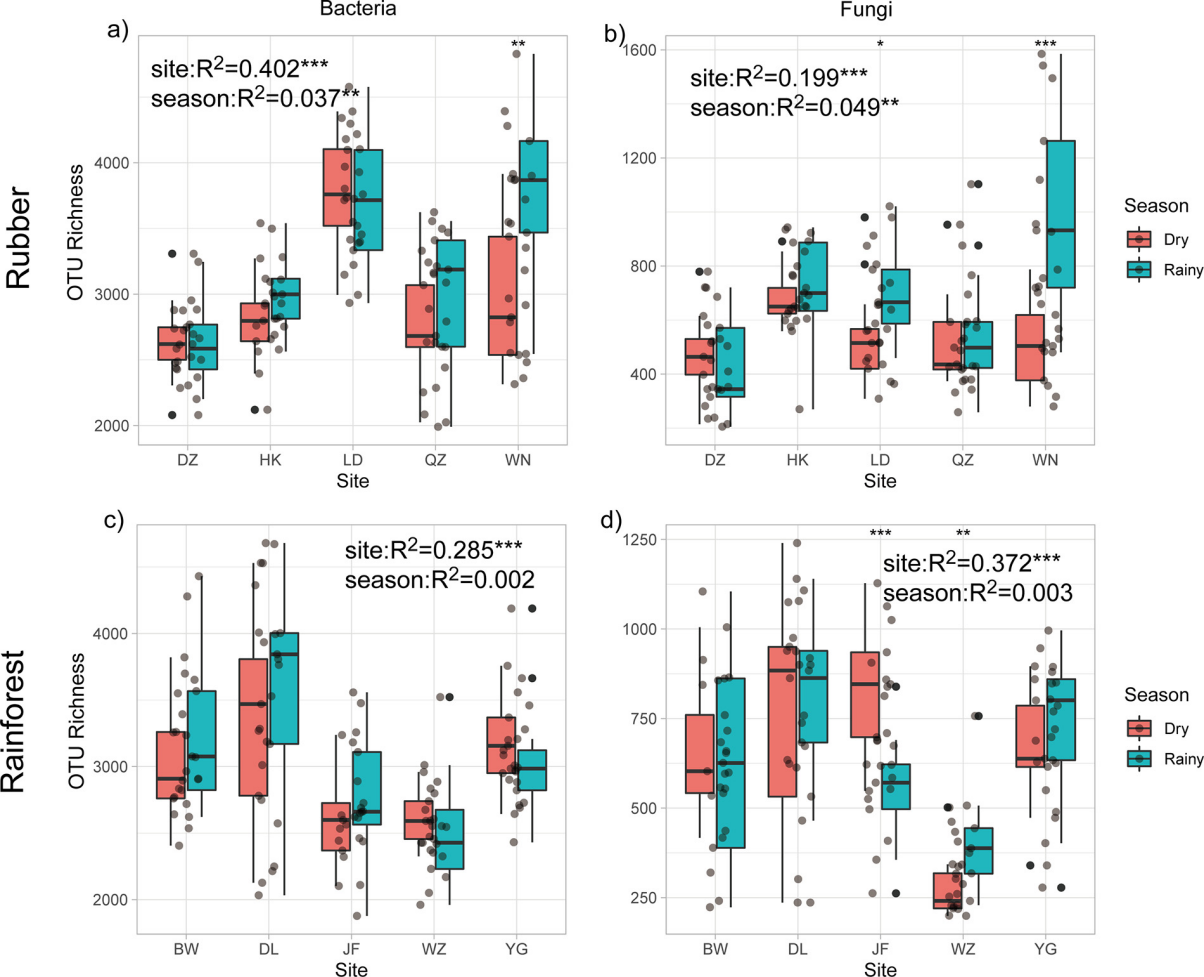
Alpha diversity of bacterial and fungal communities of rubber (a, b) and rainforest (c, d). Significant differences between seasons in each sampling area are indicated by asterisks (***, *P* < 0.01; **, *P* < 0.01; *, *P* < 0.05).

**FIG 2:**
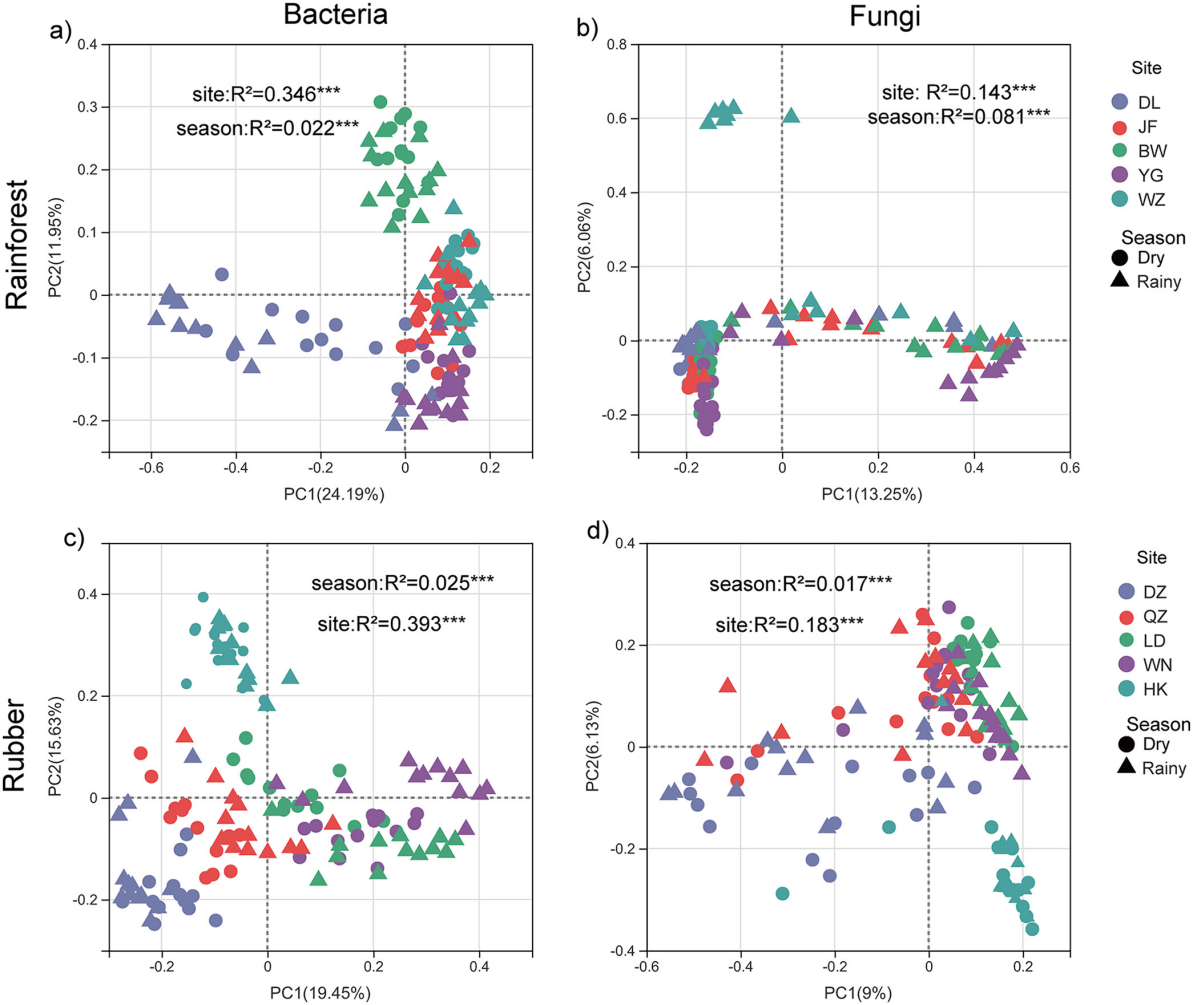
Principal coordinate analysis (PCoA) of taxonomic similarity based on Bray-Curtis distances (OTU [operational taxonomic unit] level). ***, *P* < 0.001; **, *P* < 0.01; *, *P* < 0.05.

We used the OTU richness (Sobs) index to measure microbial alpha diversity in each soil sample. Generally, season and site had significant effects on the diversity of bacterial communities. For example, season explained 3.7% and 4.9% of the variation and site explained 40.2% and 19.9% of the variation in soil bacteria and fungi, respectively, in rubber plantations (Fig. 1). For rainforest, season explained 0.2% and 0.3% of the variation and site explained 28.5% and 37.2% of the variation in soil bacteria and fungi, respectively. When considering microbial beta diversity based on the abundance-related Bray-Curtis distance, we found that for the rainforest, sampling site explained 34.6% of the bacterial variance and 14.3% of the fungal variance, while season only explained 2.2% of the bacterial variance and 8.1% of the fungal variance (Fig. 2a and b). For rubber plantation, we also found a much greater effect for site than season: 39.3% of bacterial variance and 18.3% of fungal variance were explained by site, while only 2.5% of bacterial variance and 1.7% of fungal variance were explained by season (Fig. 2c and d). Other factors explained no more than 7.0% of the variation in the soil composition at the OTU level. In brief, this study argues that site location had a stronger effect than season on both microbial alpha and beta diversity estimates.

### Drives of environmental factors in shaping soil microbial community.

**FIG 3:**
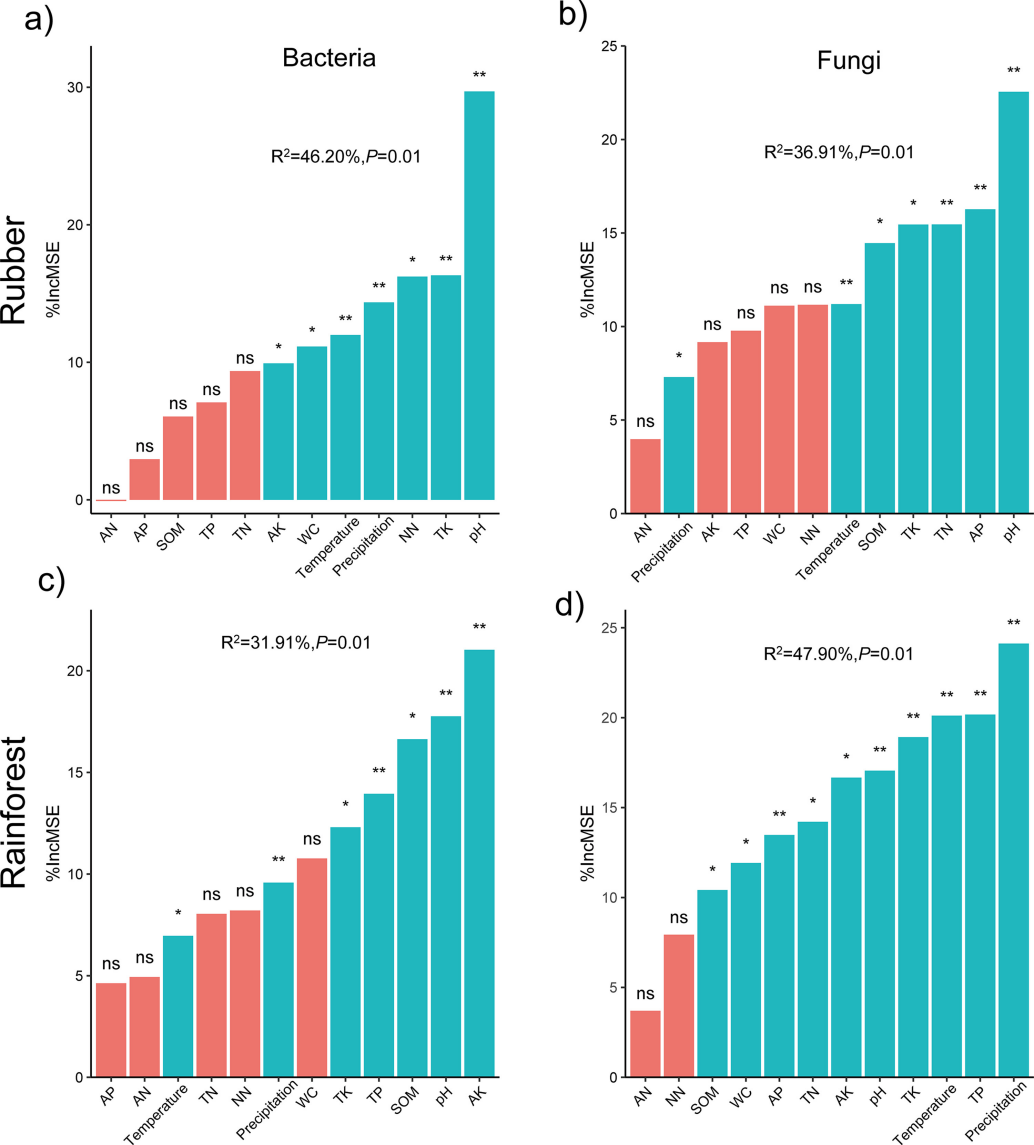
Random Forest (RF) analyses identifying the importance of potential predictors of bacterial and fungal richness. RF importance = increase in % mean square error. Blue and red columns represent *P* < 0.05 and *P* > 0.05, respectively.

**FIG 4:**
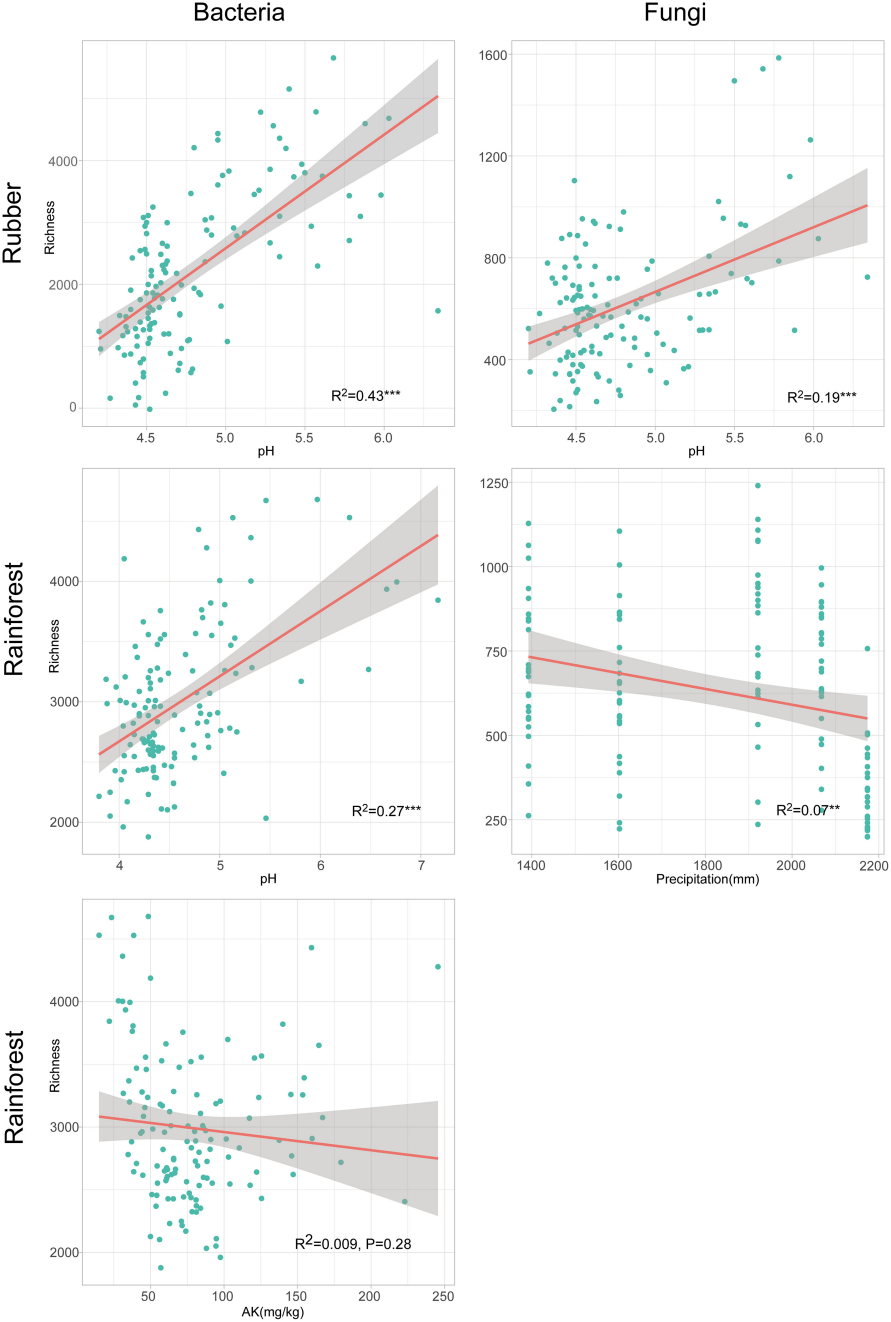
Relationships between main environmental predictors and soil microbiome richness.

Random Forest (RF) analyses indicated that soil pH was the most important predictor of bacterial richness; however, for fungal richness, pH and precipitation were the most important predictors in rubber plantations and rainforests, respectively (Fig. 3). Furthermore, these results were confirmed by significant simple linear regressions. For example, richness was strongly positively related to pH but negatively related to precipitation (Fig. 4).

**FIG 5:**
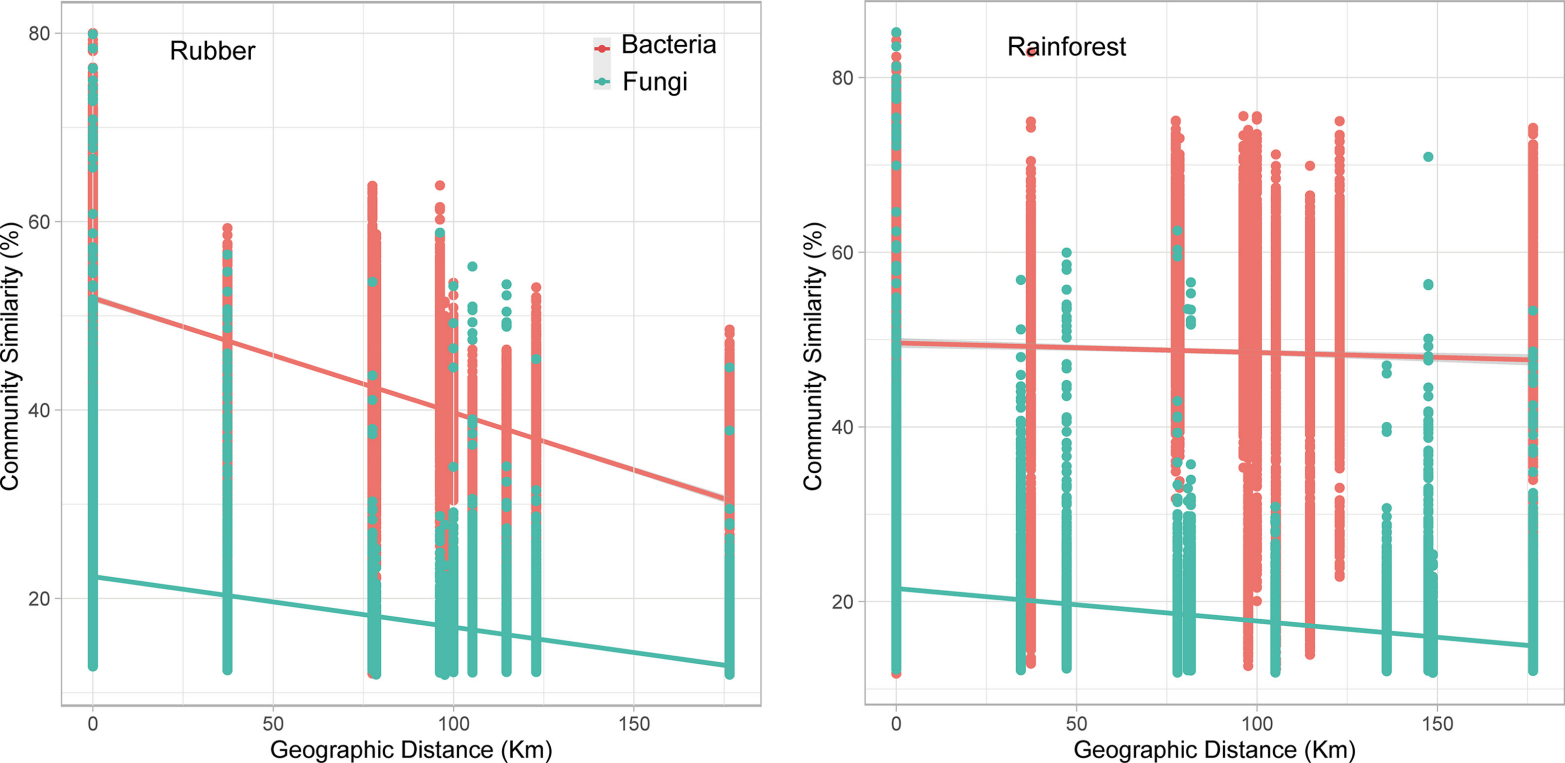
Relationships between the Bray-Curtis similarities of bacterial and fungal communities and geographic distance (OTU level).

**FIG 6:**
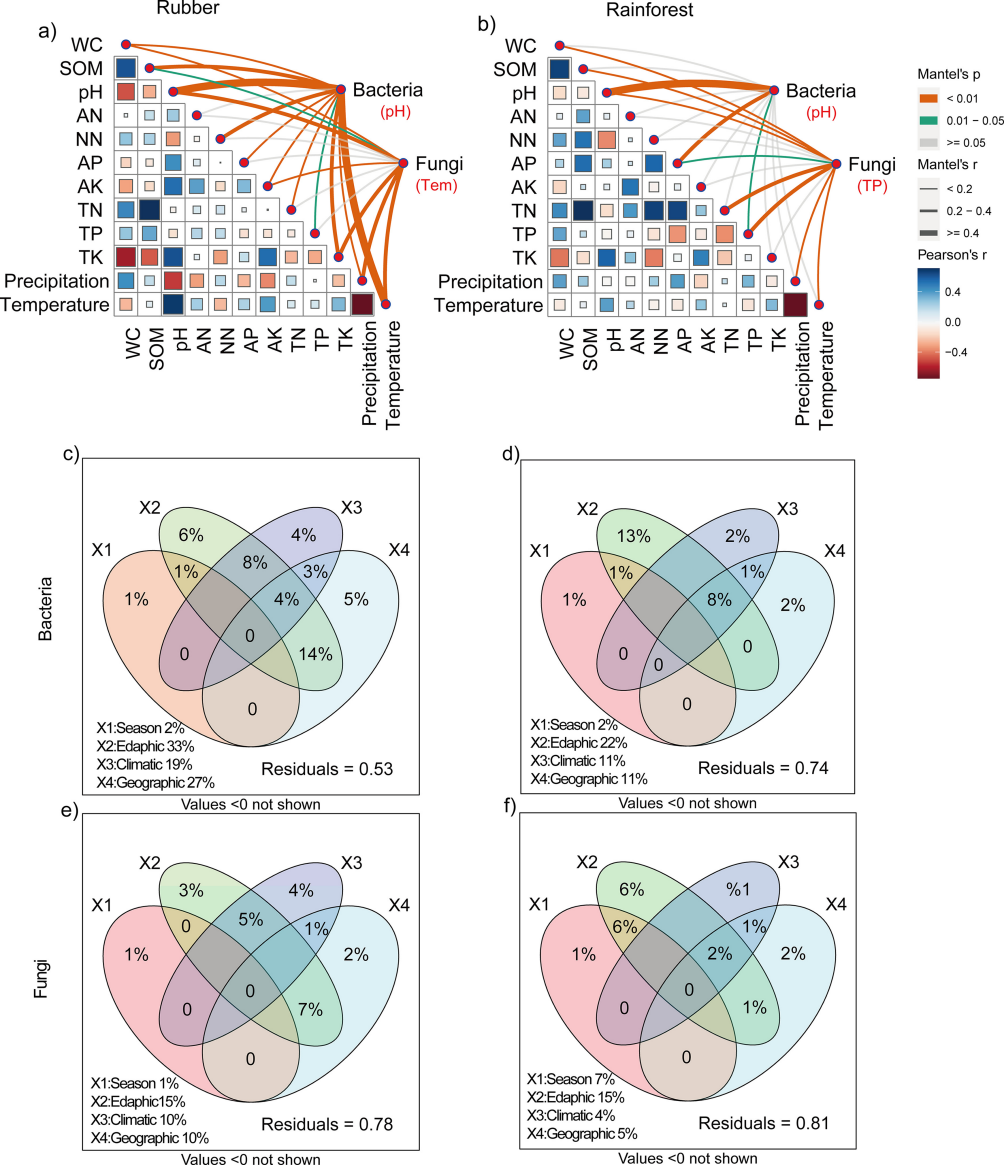
Interactions among environment, geography, and microbiotas. (a and b) Correlation analysis of edaphic properties, climatic characteristics, and microbial communities in rubber plantation and rainforest soils determined using the Mantel test. Line color represents the significance of the correlation (*P* values). Line width represents size of the correlation coefficients (Mantel’s *r*). Square color and size represent values of the correlation coefficients (Pearson’s *r*). (c to f) Variation partitioning analysis (VPA) showing the effects of edaphic properties, climatic characteristics and geographic factors, and season on microbial community composition in terms of soil beta diversity. Values indicate the percentage of variation significantly explained by each section. WC, water content; SOM, doil organic matter, pH, soil pH; AN, ammonium nitrogen; NN, nitrate nitrogen; AP, available phosphorus; AK, available potassium; TN, total nitrogen; TP, total phosphorus; TK, total potassium.

According to the Mantel test results, the environmental factors were significantly (*P = *0.001) correlated with microbial communities in fungi and bacterial communities in rubber plantations. The geographic factors were significantly related to the bacterial and fungal communities and had greater effects on the bacterial communities in rubber plantations than in rainforests (geographic distance: *r* = 0.571, environment distance: *r* = 0.447; *P* < 0.001; Table 1). These results were partially confirmed by the slopes of the distance decay relationships (DDRs; Fig. 5). Significant DDRs were observed for both the rubber plantation (bacteria: *R^2^* = 0.33, *P* < 0.001; fungi: *R^2^* = 0.16, *P* < 0.001) and rainforest (bacteria: *R^2^* = 0.0.02, *P* < 0.001; fungi: *R^2^* = 0.08, *P* < 0.001) communities. Specifically, pH was significantly correlated with bacterial community composition in each forest type, and temperature and total phosphorus (TP) were significantly correlated with fungal community composition in the rubber plantation and rainforest, respectively (Fig. 6a and b). According to a redundancy analysis (RDA), the dominant factors shaping bacterial communities in the rubber plantation and rainforest were SOM and pH (Fig. S6a and b). For the fungal communities in the rubber plantation and rainforest, an RDA showed that precipitation and TP, respectively, were the dominant drivers (Fig. S6 c and d). The relationship between the individual soil physicochemical variables and the main microbial taxa (phylum for bacteria and class for fungi) also partially supports these results (Fig. S7). We illustrated the contributions of season, edaphic, geographic, and climatic variables to bacterial and fungal community variation with a modified variation partitioning (VPA) diagram (Fig. 6). The complete set of all variables together explained 47% and 22% of the variation in the bacterial and fungal communities of the rubber plantation and 26% and 19% of the variation in the bacterial and fungal communities of the rainforest, with edaphic properties contributing the most. In addition, the VPA models revealed that factors exclusively related to climate predicted a larger significant proportion of the variation in rubber plantation communities, and the highest proportion was predicted in the fungal community (*R^2^* = 4%). Moreover, factors exclusively related to geography contributed a larger proportion of variation relative to edaphic factors to the beta diversity of rubber plantation soils (bacterial 83%, fungal 67%) than to that of rainforest soils (bacterial 15%, and fungal 33%).

**TABLE 1:** Mantel and partial Mantel test results for the correlations between community similarity and environmental and geographic distance

Factor	Controlling for	Bacteria				Fungi			
		Rubber		Rainforest		Rubber		Rainforest	
		*R*	*P*	*R*	*P*	*R*	*P*	*R*	*P*
Geographic distance		0.5711	<0.001	0.308	<0.001	0.2258	<0.001	0.18	<0.001
Environment distance		0.4467	<0.001	0.014	0.37	0.2093	<0.001	0.36	<0.001
Geographic distance	Environment distance	0.4392	0.001	0.21	0.001	0.1441	0.001	0.04	0.1
Environment distance	Geographic distance	0.2033	0.001	0.21	0.001	0.1156	0.003	0.32	0.001

## DISCUSSION

A few studies investigating tropical soil microbial communities over time at the local scale have revealed large temporal variability in their structures (4). For example, a previous study investigating soil microbial communities at the Amazon rainforest revealed high spatial heterogeneity in their structures (7); however, few studies of tropical soil microbial biogeography have been conducted to compare the effects of temporal and spatial dynamics on soil microbial community distributions on a large spatial scale. In this study, we investigated the spatiotemporal patterns of tropical soil bacterial and fungal communities across the whole of Hainan Island during the dry and rainy seasons to clarify the independent effects of space and seasonal change on microbial community variations. Our results provide solid empirical evidence that space, rather than season, explains more of the spatiotemporal variation of soil microbial communities in tropical plantations.

The effect of site location was far more important than that of seasonal change in regulating both bacterial and fungal communities at the regional scale. Previous studies have also demonstrated that space is more important than season for shaping soil microbial communities across a large spatial scale (4, 28, 29). Our results indicated that the effect of seasonal change on the microbial community composition was rather limited, yet significant (Fig. 1 and 2). Similarly, seasonal change was previously observed to have a significant effect on microbial diversity (3, 15, 30, 31). In this study, all major soil bacterial phyla and fungal classes were present in the soil samples (Fig. S5). Of the bacterial taxa, Proteobacteria and Acidobacteria encompassed the largest proportion of sequences, while Agaricomycetes and Sordariomycetes were the most abundant fungal class. The major microbial taxa are sensitive to mean annual precipitation and soil pH according to their life strategies; for example, the Proteobacteria have been assigned as copiotrophic bacteria and the Acidobacteria are strongly linked to pH. Therefore, it is not surprising to find that these groups responded to altered edaphic and climatic factors (Fig. S7), as has already been demonstrated (32–34). In the present study, environmental dissimilarity significantly increased with geographic distance. Furthermore, we observed a stronger effect for geographic location than for season upon environmental variables. Essentially, the dominant drivers of microbial communities, for example, soil edaphic (e.g., soil pH) and climatic factors (e.g., precipitation), were mainly affected by geographic location, thus contributing to the mechanisms driving microbial spatial variation.

We also uncovered evidence indicating that edaphic variables underpin the mechanisms responsible for soil microbial variation in Hainan Island. Among the edaphic variables, we found that soil pH was the dominant predictor for bacterial richness, as has also been found in numerous previous studies (35–39), and bacterial richness was positively correlated with pH in both the rubber plantation and rainforest. Likewise, pH was a dominant driver of bacterial communities in the tropical forest because bacterial communities are most sensitive to soil pH (40, 41). For fungal richness, soil pH and MAP were the most important predictors in the rubber plantation and rainforest; this result was consistent with that of a previous study on a global scale (6). Compared with bacterial communities, fungal beta diversity was affected by climatic (MAT and MAP) and edaphic variables (TP) in rubber plantation and rainforest ecosystems, respectively. The weaker geographic effect on fungal communities coupled with the effects of distinct dominant factors on fungal beta diversity between the two forests (Fig. 4) could be linked to the fact that fungi have weak stability and resistance to the external environment (38, 41) and greater individual body sizes than bacteria (40, 42, 43), while bacteria have greater motility than fungi (44, 45). In addition, taxa belonging to Tremellomycetes (Fig. S5), which produce large quantities of aerially dispersed mitospores, were generally widely distributed, which could lend further support to these results (6). It is worth noting that edaphic factors evidently affected microbial community similarity, suggesting that deterministic processes drive community assembly. Geographic factors contributed a larger proportion of variation relative to edaphic factors to the beta diversity of rubber plantation soils than to that of rainforest soils, indicating a stronger effect of stochastic processes in driving the beta diversity of rubber plantation soils. This result was also confirmed by the VPA models and Mantel test. This is also partially consistent with previous studies (46).

### Conclusions.

The soil microbial communities of the two forests were not only affected by environmental factors (e.g., edaphic and climatic factors), but also by different dominant geographic factors. In particular, our work showed that spatial variation in bacterial and fungal community composition was mainly dominated by edaphic properties (e.g., pH) and climatic factors (e.g., MAT and MAP). Moreover, the environmental factors were mainly explained by geographic location rather than by season, and environmental dissimilarity significantly increased with geographic distance. In conclusion, our study provides solid empirical evidence that space, rather than season, explained more of the spatiotemporal variation of soil microbial communities in the tropical forest.

## MATERIALS AND METHODS

### Study site and sampling.

The study was conducted on Hainan Island (18°10′ to 20°10′N and 108°37′ to 111° 03′E), China. Hainan has a warm and humid climate all year round, with evident dry (November to April) and rainy (May to October) seasons within a year. Rubber forest plantations are mainly distributed on the platforms around the central mountainous areas with convenient transportation and nearby water sources. The tropical rainforest is mainly distributed in the central part of the mountains.

The soil sampling approach described here is based on that of our previous study (5). Sampling sites are shown in Fig. S1 in the supplemental material. From each site, 13 repetitions were sampled as previously described (5). Information on our study sites is presented in Table S1. Each site had 13 soil samples and there were 65 samples. Sample collections in these sites were performed in January and July of 2018, corresponding to the dry and rainy seasons, respectively. Thus, we obtained a total of 260 soil samples (including rubber plantation and rainforest). Each sample was then divided into two parts: one for analysis of soil physicochemical characteristics, and the other to be stored at −80°C for future analyses (DNA extraction). Soil physicochemical characteristics were analyzed using standard soil test methods described by Lu (47). More detailed descriptions of the methods for soil properties measurement are described by Lan et al. (5) and provided in the supplemental materials (Methods S1).

### DNA extraction and Illumina sequencing.

Microbial DNA was extracted from 0.5 g of soil using the E.Z.N.A. Soil DNA kit (Omega Bio-Tek Inc., Norcross, GA, USA) following the manufacturer’s protocols. The primer pair ITS1F (5′-CTTGGTCATTTAGAGGAAGTAA-3′) and ITS2R (5′-GCTGCGTTCTTCATCGATGC-3′) was used to amplify the fungal ITS1 region (48). The pair 515FmodF (5′-GTGYCAGCMGCCGCGGTAA-3′) and 806RmodR (5′-GGACTACNVGGGTWTCTAAT-3′) was used to amplify the 16S V4 hypervariable region (49, 50). Sequencing was performed on the Illumina MiSeq platform according to standard protocols.

### Data analysis.

Raw .fastq files were demultiplexed and quality-filtered using QIIME (version 1.9.1) (51). Both the alpha diversity and beta diversity of the fungal community were calculated in QIIME. OTUs were clustered with 97% similarity cutoff using UPARSE (52) (version 7.1) and chimeric sequences were identified and removed using UCHIME. The taxonomy of each OTU representative sequence was analyzed by RDP Classifier version 2.2 (53) against the 16S rRNA and 18S rRNA databases using a confidence threshold of 0.7 (17). We modeled the alpha diversity (expressed as observed richness) as a response variable and site location and seasonal change as fixed effects. Pairwise comparison results were obtained using the Wilcoxon test with the *P* values corrected using the false discovery rate method.

Random Forest analysis was used to identify the main environmental drivers for soil fungal alpha diversity (54, 55). In the RF models, edaphic (soil pH, WC, SOM, TK, TN [total nitrogen], TP, AN, NN, AK, AP [available phosphorus]) and climatic (temperature and precipitation) properties served as predictors for soil microbial richness. To estimate the importance of these variables, we used percentage increases in the mean squared error (MSE) of variables: higher MSE% values imply more important variables. The “vegan” package was used to calculate Bray-Curtis similarity matrices for bacterial and fungal community data. Distance-decay relationships were estimated by Bray-Curtis similarity and geographic distance. We carried out standard and partial Mantel tests on the Bray-Curtis distances and geographic distances of significant variables to determine the effects of geographic distance and environmental variables on microbial communities. Pairwise geographic distances between samples were calculated using the “geosphere” R package with the latitude and longitude coordinates. We implemented a principal coordinate analysis (PCoA) based on Bray-Curtis distances for the 130 samples to explore bacterial and fungal community composition differences. Differences in community composition between the investigated site locations and seasonal changes were tested using permutational multivariate analysis of variance analysis in R “vegan” with 999 permutations. Latitudinal and longitudinal data for each site were transformed to rectangular data to represent spatial distance by function pcnm, and variation partitioning analyses were conducted with function varpart in the “vegan” package for R. We used variation partitioning (56) to quantify the relative importance of seasonal change (dry and rainy season), climatic factors (temperature and precipitation), edaphic factors (including soil pH, WC, SOM, TK, TN, TP, AN, NN, AK, AP), and geographic variables.

### Data availability.

The raw reads were deposited into the NCBI Sequence Read Archive database (accession no.: SRP278296, SRP278319).
