# Supplementary material for: Space Rather than Seasonal Changes Explained More of the Spatiotemporal Variation of Tropical Soil Microbial Communities
Source: Microbiol Spectr. 2022 Nov 23;10(6):e01846-22. doi: 10.1128/spectrum.01846-22 (PMC9769686; doi:10.1128/spectrum.01846-22)
Supplement: Supplemental file 1 — Supplemental material. Download spectrum.01846-22-s0001.pdf, PDF file, 0.9 MB [file spectrum.01846-22-s0001.pdf]

SUPPORTING INFORMATION

Space rather than seasonal changes explained more of the spatiotemporal variation of tropic soil microbial communities

Yaqing Wei[a,c,d#], Fei Quan[b,c#], Guoyu Lan[b,c*], Zhixiang Wu[b,c], Chuan Yang[b,c]

The following Supporting Information is available for this article:

Method S1

Soil samples were analyzed using standard soil test methods described by Lu (1). Soil pH was measured in a 1:1 soil: water mixture. Soil moisture was measured gravimetrically. Soil total nitrogen (TN) was determined using micro-Kjeldahl digestion followed by steam distillation. Total phosphorus (TP) and total potassium (TK) were measured following digestion with NaOH. Nitrate nitrogen (NN) and ammonium nitrogen (AN) were determined by steam distillation and indophenol-blue colorimetry, respectively. Soil samples were extracted with NaHCO3 and the extract was then used to measure available soil phosphorus (AP) via molybdate-blue colorimetry. For determination of available soil potassium (AK), soil was extracted with ammoniumacetate and then the extract was loaded onto an atomic absorption spectrometer with ascorbic acid as a reductant (2).

Table S1 Site information on rubber plantations and tropical rainforest.

**Forest**	**Site**	**Longitude**	**Latitude**	**Elevation （m）**	**Mean annual precipitation (mm)**	**Mean annual temperature (℃)**
Rubber plantation	Danzhou (DZ)	109.5789	19.5628	112	1831.53	23.60
Rubber plantation	Qiongzhong (QZ)	109.7387	19.2633	156	2067.33	23.45
Rubber plantation	Ledong (LD)	109.2231	18.7534	170	1661.26	24.46
Rubber plantation	Wanning (WN)	110.132	18.6749	51	1786.46	24.7
Rubber plantation	Haikou (HK)	110.5723	19.6977	102	1863.38	24.16
Rainforest	Diaoluoshan (DL)	109.864	18.7277	958	1921.27	24.17
Rainforest	Jianfengling (JF)	108.8834	18.7273	950	1392.34	24.69
Rainforest	Bawangling (BW)	109.1277	19.0842	575	1602.12	24.31
Rainforest	Yinggeling (YG)	109.5598	19.0467	620	2067.77	23.55
Rainforest	Wuzhishan (WZ)	109.6812	18.9059	820	3173.82	23.42

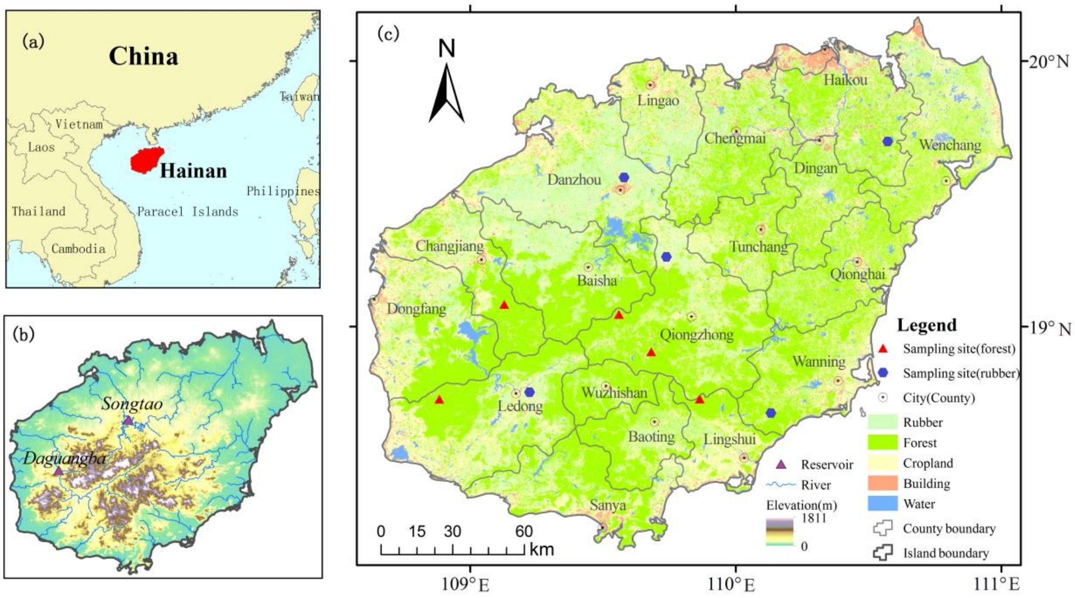

Figure S1 Study sites of the rainforest and rubber plantation of Hainan Island. Blue solid hexagon: rubber plantations.

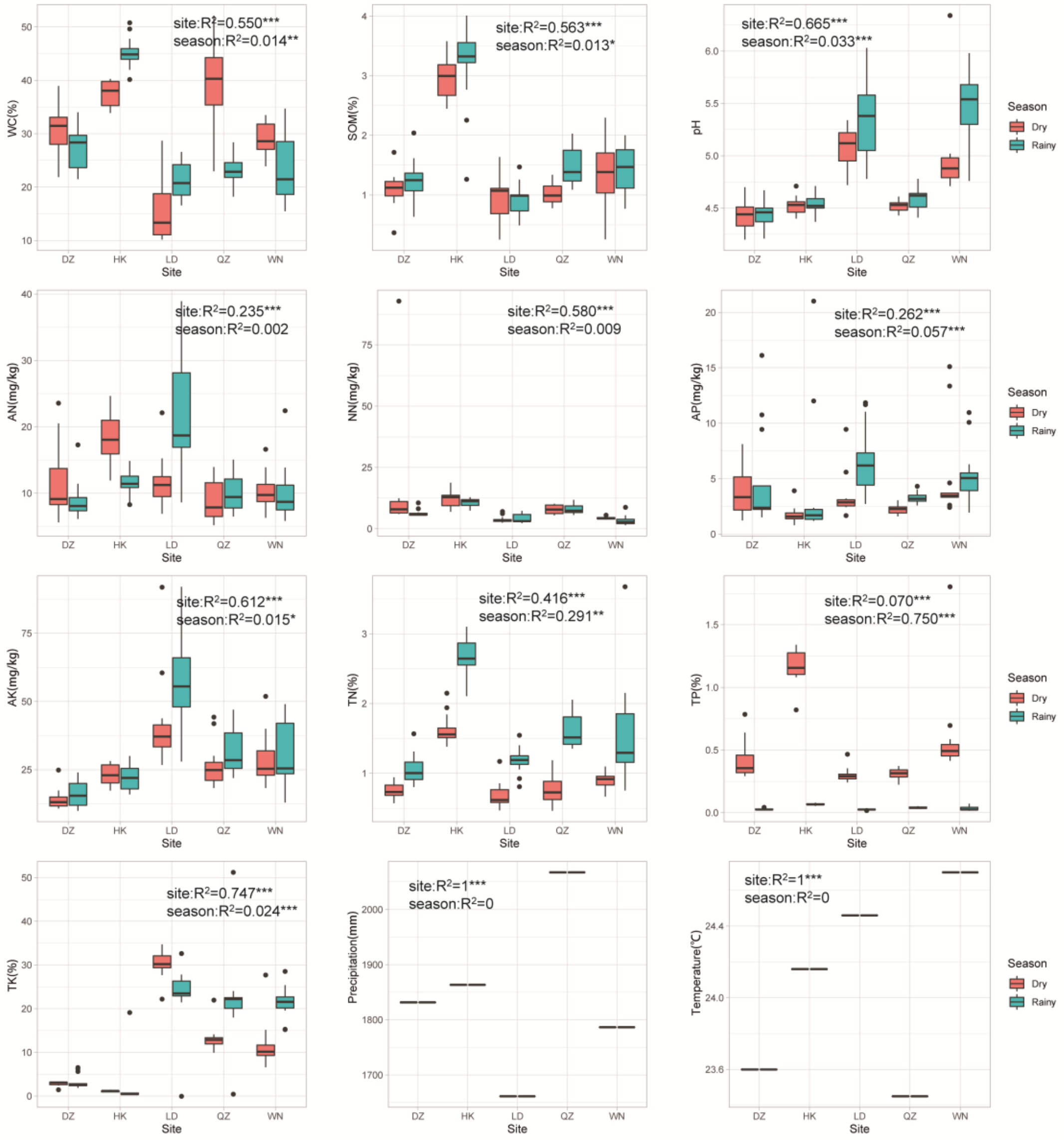

Figure S2 Environmental variables in dry and rainy seasons among the 5 sampling sites of rubber plantation. The significant differences between seasons and among sites were detected by adonis. SOM: Soil organic matter, TN: Total nitrogen, TP: Total phosphorus, TK: Total potassium, WC: Water content, pH: Soil pH. AN: Ammonium nitrogen, NN: Nitrate nitrogen AP: Available phosphorus, AK: Available potassium. *, $P < 0.05$; **, $P < 0.01$, ***, $P < 0.001$

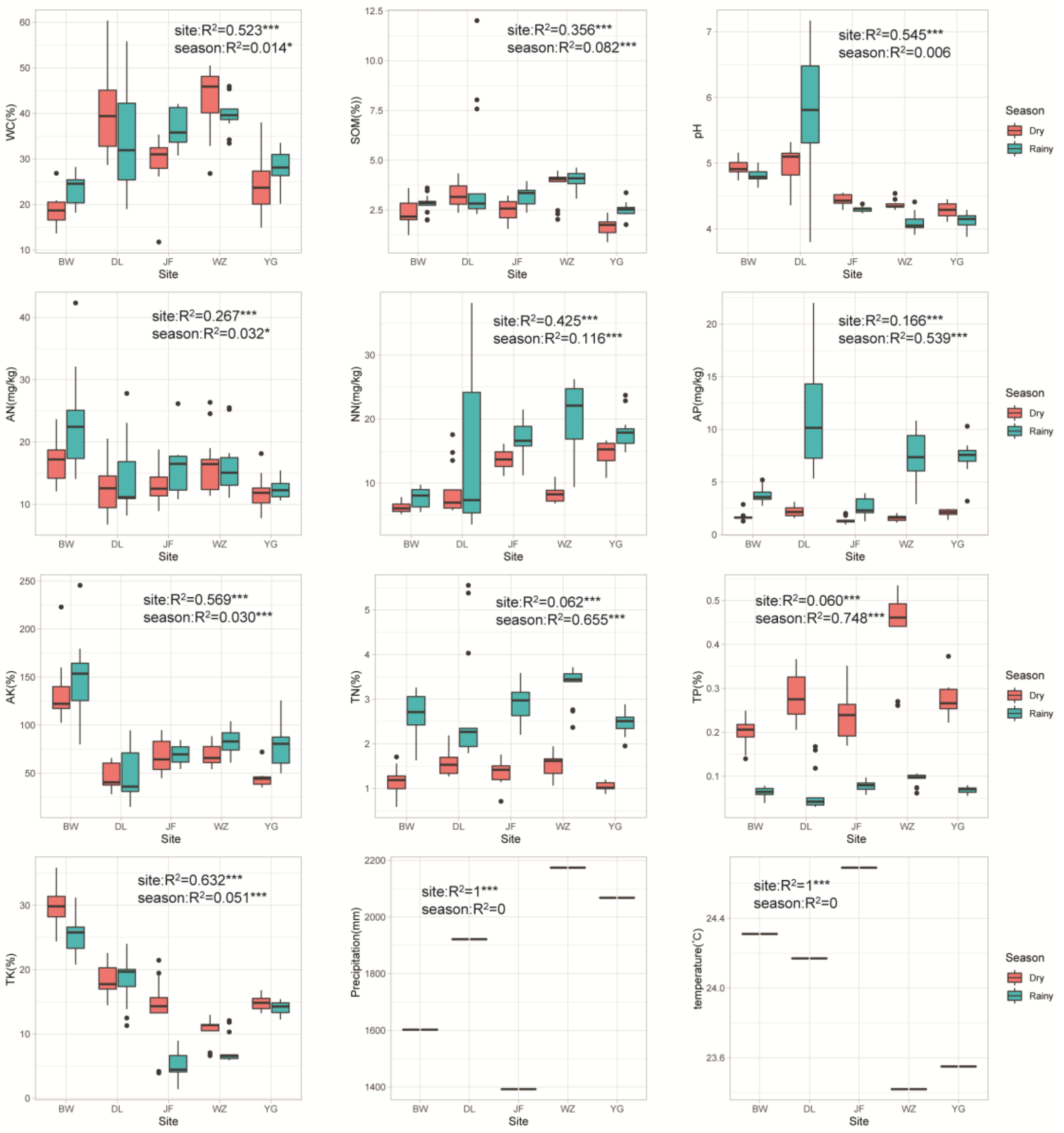

Figure S3 Environmental variables in dry and rainy seasons among the 5 sampling sites of rainforest. The significant differences between seasons and among sites were detected by adonis. SOM: Soil organic matter, TN: Total nitrogen, TP: Total phosphorus, TK: Total potassium, WC: Water content, pH: Soil pH. AN: Ammonium nitrogen, NN: Nitrate nitrogen AP: Available phosphorus, AK: Available potassium. *, $P < 0.05$; **, $P < 0.01$, ***, $P < 0.001$.

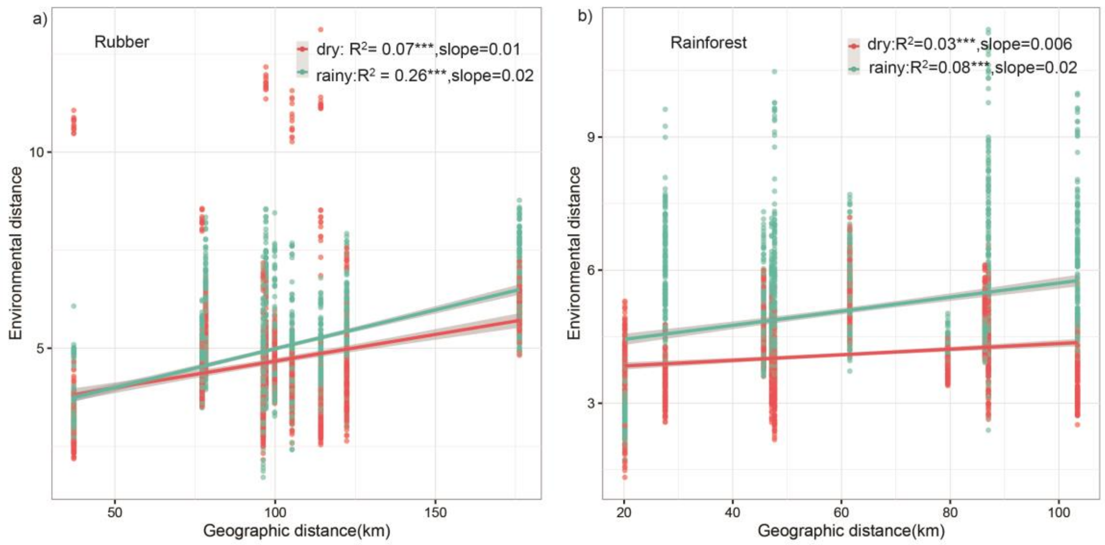

Figure S4 Relationship between geographic distance and environmental distance based on Euclidean distance.

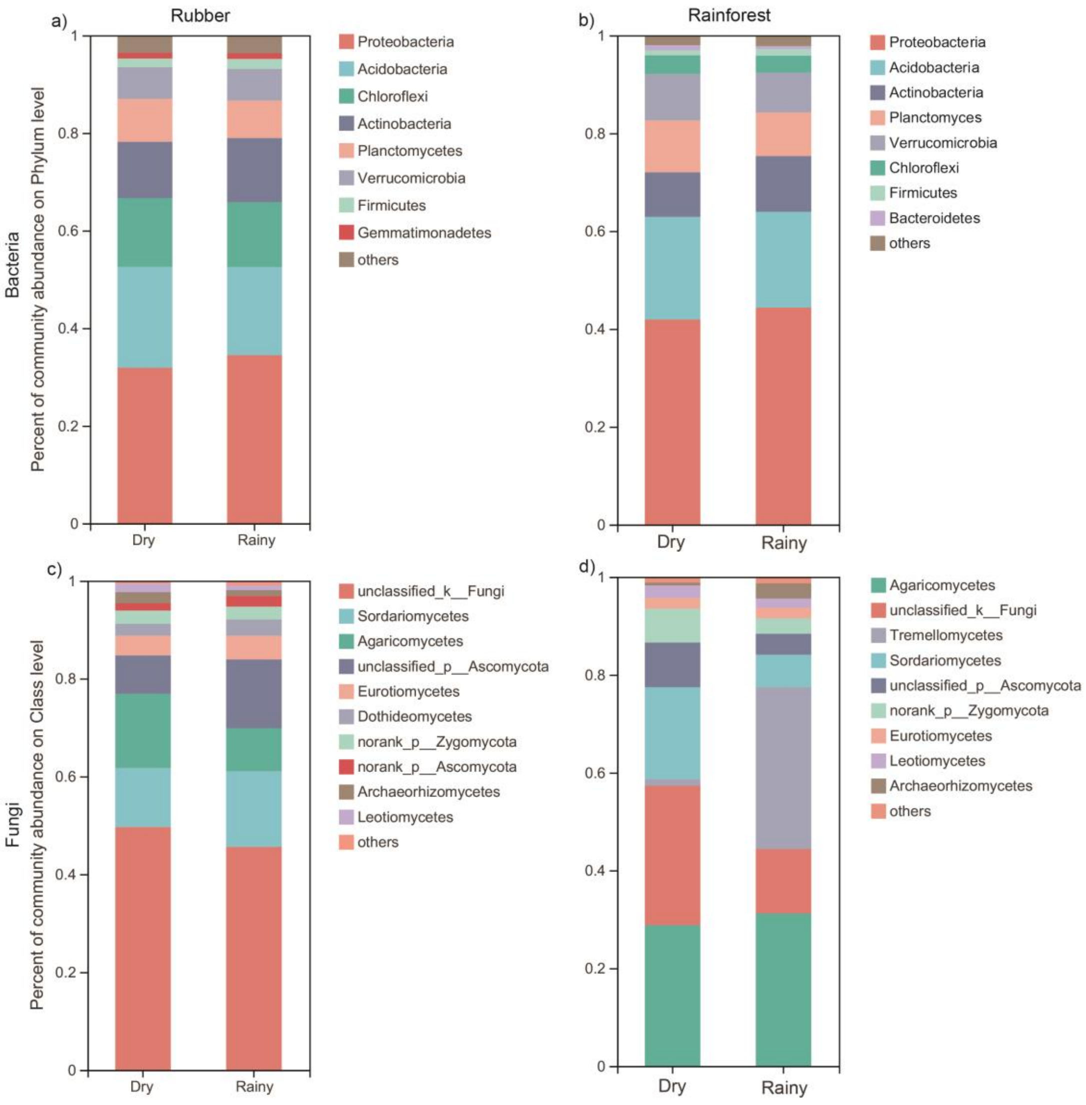

Figure S5 Stacked bars showing the relative abundance of bacterial phyla (a), (b) and fungal classes (c), (d) in the dry and rainy seasons.

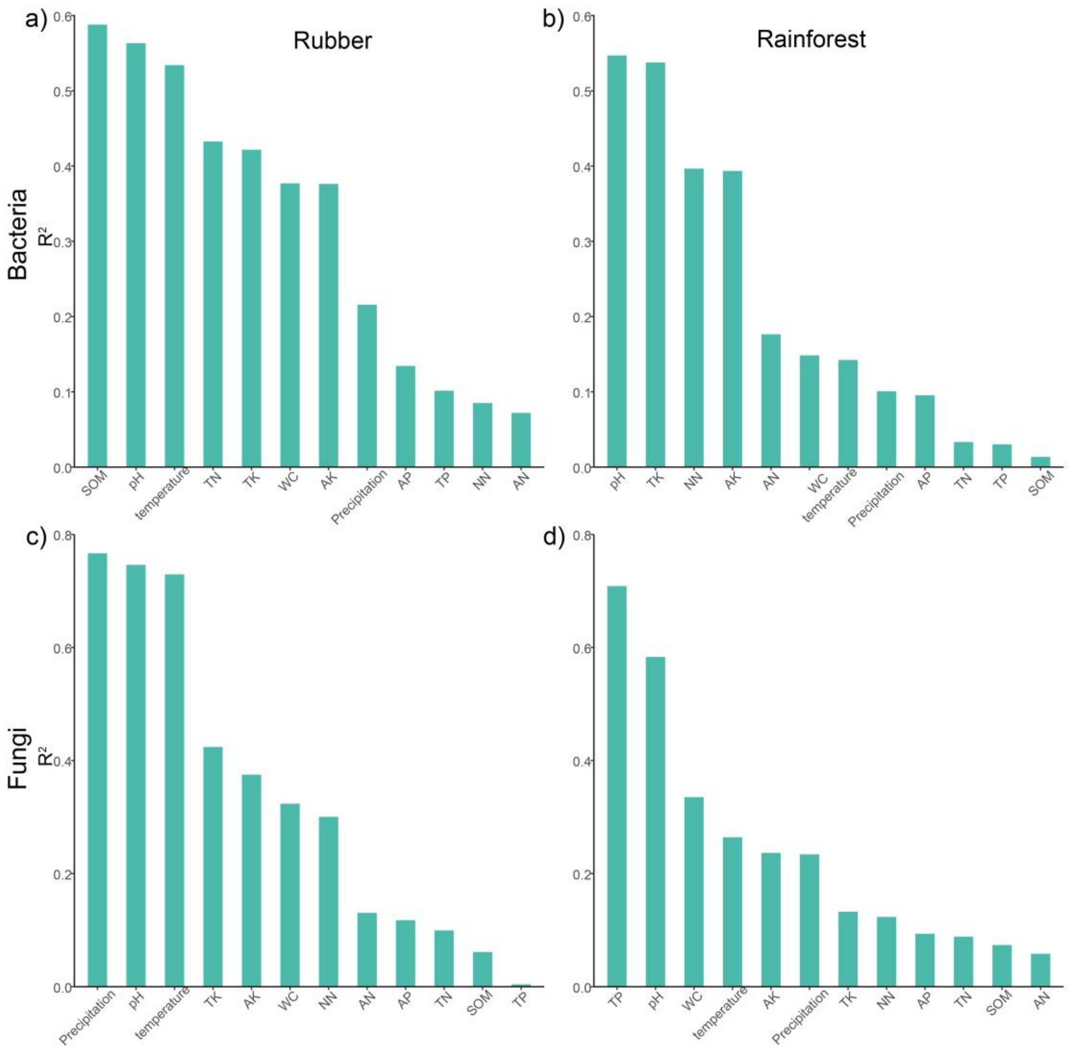

Figure S6 Redundancy analysis ordination of the soil samples, based on the fungal community composition (OTU level) of rubber plantation and tropical rainforest sites. SOM: soil organic matter, TN: total nitrogen, TP: total phosphorus, TK: total potassium, WC: water content, pH: soil pH, AN: ammonium nitrogen, NN: nitrate nitrogen AP: available phosphorus, AK: available potassium.

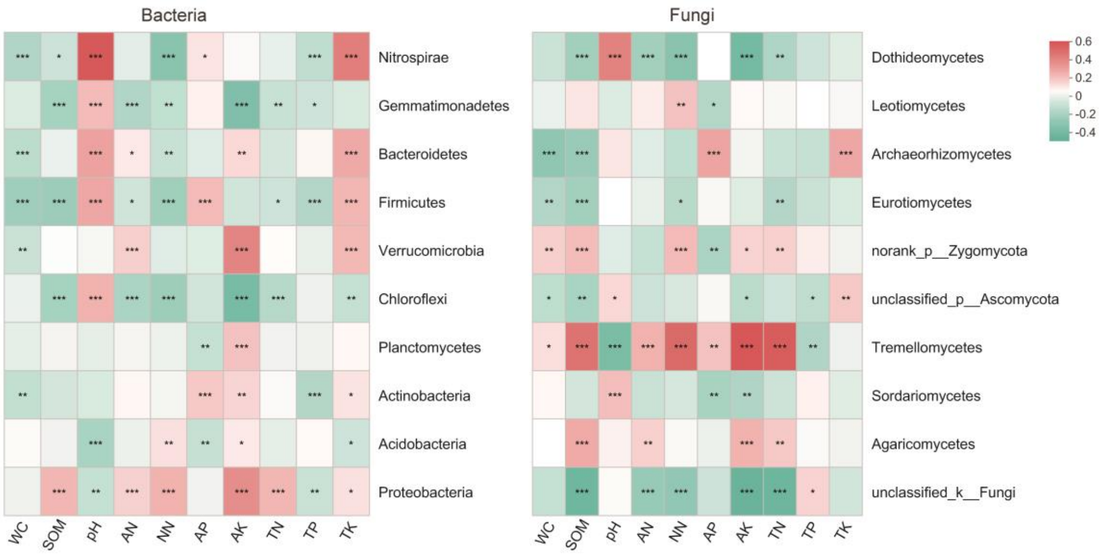

Figure S7 Spearman correlation heatmap of bacterial (at the phylum level) and fungal (at the class level) community composition versus the soil and environmental factors. Colors indicate the Spearman correlation coefficients, as shown in the key. SOM: soil organic matter, TN: total nitrogen, TP: total phosphorus, TK: total potassium, WC: water content, pH: soil pH, AN: ammonium nitrogen, NN: nitrate nitrogen AP: available phosphorus, AK: available potassium.

REFERENCES

1. Lu, R.K., 1999. Methods for Soil and Agriculture Chemistry Analysis. Chinese Agricultural Science and Technology Press, Beijing, pp. 20–35.
2. Chen J, Xu H, He D, Li Y, Luo T, Yang H, Lin M. 2019. Historical logging alters soil fungal community composition and network in a tropical rainforest. Forest Ecology and Management 433:228-239.
